# Combating Rhino Horn Trafficking: The Need to Disrupt Criminal Networks

**DOI:** 10.1371/journal.pone.0167040

**Published:** 2016-11-21

**Authors:** Timothy C. Haas, Sam M. Ferreira

**Affiliations:** 1 Lubar School of Business, University of Wisconsin-Milwaukee, Milwaukee, Wisconsin, United States of America; 2 Scientific Services, SANParks, Skukuza, South Africa; University of Lleida, SPAIN

## Abstract

The onslaught on the World’s wildlife continues despite numerous initiatives aimed at curbing it. We build a model that integrates rhino horn trade with rhino population dynamics in order to evaluate the impact of various management policies on rhino sustainability. In our model, an agent-based sub-model of horn trade from the poaching event up through a purchase of rhino horn in Asia impacts rhino abundance. A data-validated, individual-based sub-model of the rhino population of South Africa provides these abundance values. We evaluate policies that consist of different combinations of legal trade initiatives, demand reduction marketing campaigns, increased anti-poaching measures within protected areas, and transnational policing initiatives aimed at disrupting those criminal syndicates engaged in horn trafficking. Simulation runs of our model over the next 35 years produces a sustainable rhino population under only one management policy. This policy includes both a transnational policing effort aimed at dismantling those criminal networks engaged in rhino horn trafficking—coupled with increases in legal economic opportunities for people living next to protected areas where rhinos live. This multi-faceted approach should be the focus of the international debate on strategies to combat the current slaughter of rhino rather than the binary debate about whether rhino horn trade should be legalized. This approach to the evaluation of wildlife management policies may be useful to apply to other species threatened by wildlife trafficking.

## Introduction

Unprecedented wildlife trafficking threatens biodiversity [1]. Asians consume charismatic mammal products often obtained from non-Asian sources [2]. There is some evidence that demand for rhino horn among Asian consumers is inelastic [3]. This means that the quantity of rhino horn demanded by consumers does not change when the price of such horn changes [4]. Inelastic demand contributes to a perception of high profit potential [5] in rhino poaching, particularly by organized crime syndicates. Often such syndicates recruit individuals living in communities adjacent to natural parks and private game reserves, especially if these protected areas have populations of rhinos. Attractiveness of illegal activities rises when communities have historically traded in or used wildlife products, but now wrestle with unclear rights over wildlife access and experience intense and uncompensated human-wildlife conflicts [5]. When anti-poaching enforcement is inadequate, organized crime provides opportunities for some community members to resolve these issues to their own benefit by engaging in the resultant low-risk activity of poaching. Such poaching quickly evolves into a supply chain that is monopolized by organized crime syndicates [6]. Governance systems that administer these communities and the adjacent protected areas incur much of the blame for such increased poaching activity. They do so by not resolving wildlife ownership questions, leaving community members uncompensated for any conflicts with wildlife, and failing to enforce anti-poaching laws [7].

For rhinos, reducing demand and providing supply [8] offer policies to combat horn trafficking that target economic drivers. Advocates of no-trading in rhino horn [9–11] see demand reduction as the key policy response. Promoters of legal trading in rhino horn [12–14] assume that providing legal supply can meet demand and displace illegal rhino horn trade. Even so, models suggest that without consumer behavior modification, legal trade in rhino horn leads to extinction [15]. In our Discussion, below, we review the evidence that leads to the same conclusion for the related question of whether the trade in elephant ivory should be legalized. There, we also review the general conditions for the coexistence of legal and black markets in a commodity.

The combination of governance failures and the attractiveness of wildlife trafficking facilitate organized crime entering the illegal supply chain [16]–with attendant increases in poaching [17]. Because organized crime syndicates control middlemen, once these syndicates add wildlife trafficking to their roster of illegal activities, they tend to monopolize that trade. Then, any attempt to conduct legal trade in a wildlife product is jeopardized. This is because either the syndicate itself will purchase such legally harvested wildlife products with the intention of reselling them; or will lower their own prices for the product. Such temporary or permanent price reductions are not difficult for criminal syndicates due to their diversified business model that tolerates a wide range of profit margins, low overheads and large cash reserves [18]. Either way, price conscious consumers will see little reason to purchase legal as opposed to illegally harvested products.

Demand reduction campaigns are also jeopardized by these same criminal syndicates. Syndicates have the ability to adjust to interventions by authorities [19] and again, can modify their prices as necessary to maintain sales volume. Specifically, a price reduction may be needed when the attractiveness of the wildlife product to consumers targeted by the demand reduction campaign is reduced—thereby reducing the reserve price of these consumers. Organized crime thus has the ability to undermine both trade and consumer behavior modification policies.

Because of the transnational nature of an organized crime network involved in wildlife trafficking, regionally- and nationally-authorized police forces are limited in their ability to track, arrest, and prosecute many of the network’s middlemen. In particular, the distributed and resilient management structure of these criminal syndicates makes them difficult to shutdown unless international, coordinated investigations are under taken to identify and prosecute higher-tier syndicate members [19–20]. Such a transnational policing effort would take considerable manpower, money, and high levels of cooperation between countries. This is because intelligence data would need to be gathered and shared across regions and countries; and arrest warrants, extraditions, and banking interventions would need to be arranged by cooperating authorities across several countries. Some guidance is given in [20] on how to effectively conduct such a transnational policing effort. Because of the trafficking syndicate’s international extent however, expansion of anti-poaching operations within rhino-hosting protected areas needs to be coordinated with increased efforts towards disrupting the criminal network at levels higher than the on-the-ground poacher. The management policies we study here clearly separate within-reserve anti-poaching efforts from criminal network disruption efforts.

People that live next to protected areas struggle with unclear property rights and human-wildlife conflict disincentives created by wildlife conservation policies developed by elites with little input from indigenous communities [5, 21]. Trafficking syndicates exploit this frustration in order to recruit some of these people for poaching expeditions. There is some evidence however, that providing legal economic opportunities to people living next to parks reduces their interest in poaching [22].

We hypothesize then that a policy of disrupting horn trafficking syndicates combined with addressing the inequality rife in communities living next to protected areas [23] could lead to rhino sustainability. Hence, this policy should be considered as an attractive option regardless of whether authorities favor demand reduction, asset protection, or supply provision policies. Here, we evaluate this hypothesis by constructing a model composed of an agent-based economic sub-model that interacts with an individual-based ecological sub-model. We use this *economic-ecological model* to evaluate the impact on rhino abundance of various management policies. These policies are mixes of (a) criminal network disruption complimented by increased legal economic opportunities for people living next to parks; (b) legal trade in rhino horn; (c) increased within-reserve anti-poaching efforts; and (d) demand reduction media campaigns.

## Materials and Methods

### Study population

Our focus is on southern white rhinos (*Ceratotherium simum simum*) in South Africa. During 2012, South Africa was home to approximately 21,000 of the World’s 25,500 African rhinos (black and white) [17]. A total of 92.7% of the global population of white rhinos resided in South Africa. White rhinos occur in various land uses and designations. National Parks (4 Parks) protected 56.4%, Provincial Reserves (36 Reserves) 19.6% and Private Reserves (≈420 Reserves) 24.0% of South Africa’s white rhinos during 2012 [17]. No white rhinos were owned or formed part of custodianships assigned to traditional communities that own land living next to protected areas.

Kruger National Park (KNP) is South Africa’s key Park providing protection to 55.6% of South Africa’s white rhinos [24]. During 2013, block sample counts estimated that between 8,394 to 9,564 white rhinos lived in KNP [25]. Most concerning was that recorded birth rates (7%–8.5%) equaled or were slightly less than loss rates that comprised poaching (6%–6.5%), natural mortality (1%–2%) and management removals (<0.5%) [25]. Poaching pressure, however, was uneven across the KNP landscape with some areas remaining relatively free of poaching incidences. The areas with highest white rhino densities had relatively few poaching incidences with resultant negligible effects on the local population. In these areas, density and environmental factors reduce white rhino birth rates and increase natural mortality.

### Data collection

Data used to validate our economic-ecological model described below includes prices paid to poachers at various levels of the supply chain. Authorities define five levels in the illegal supply chain [26]. Members of Level 1 are poachers who typically organize themselves into teams of three people as they enter protected areas to hunt for rhino and poach them. A Level 1 poacher is paid per hunt. Members of Level 2 are transporters who are paid per transporting event. Members of Level 3 are local middlemen who often oversee a number of poaching teams and transporters. Middlemen get paid per kilogram of horn. Members of Level 4 are exporters who transport rhino horns via airports or harbors to destinations in Asia. Exporters receive payment per item. Finally, members of Level 5 are traders who sell trafficked rhino horn directly to consumers in end-user states. We collated prices paid at different levels and how these changed from the interview records of captured poachers as well as intelligence gathered by investigators (Table 1). We acknowledge, however, that some uncertainty remains. To assist in defining trends in potential consumers, we extracted Asian population projections up to 2040 (Table 2). We used population changes from 2006 to 2014 contained in this data to parameterize the agent-based economic sub-model [27].

**Table 1:** Prices paid to participants at various levels of illegal rhino horn supply chains from 2006 to 2014.

	Poacher						Transporter	Middleman	Exporter		Trader	
	Paid per hunt (Up to April 2014)			Paid per kilogram(Since April 2014)			Paid per event	Paid perkilogram	Paid per item		Mark up perkilogram	
	WaterCarrier	AxeCarrier	Shooter	WaterCarrier	AxeCarrier	Shooter			Airport	Harbor	Vietnam	China
2006	$89–356	$89–356	$89–356	-	-	-	Level 1poachers were also transporters	$3703–5185	Nodata	Nodata	No data	Nodata
2007	No data	Nodata	Nodata	-	-	-	Level 1poachers werealsotransporters	No data	Nodata	Nodata	No data	Nodata
2008	$194–607	$194–607	$583–1822	-	-	-	Level 1poachers werealsotransporters	$4252–6317	Nodata	Nodata	No data	Nodata
2009	No data	Nodata	Nodata	-	-	-	Level 1poachers were also transporters	No data	Nodata	Nodata	No data	Nodata
2010	$548–1096	$548–1096	$1644–3287	-	-	-	Level 1poachers werealsotransporters	$5479–8903	Nodata	Nodata	No data	Nodata
2011	No data	Nodata	Nodata	-	-	-	Level 1poachers werealsotransporters	No data	Nodata	Nodata	No data	Nodata
2012	$488–2074	$488–2074	$1464–6222	-	-	-	$488–976	$9149–18299	Nodata	Nodata	600%	75%
2013	$830–1661	$830–1661	$2491–4982	-	-	-	$415–830	$6227–12455	Nodata	Nodata	600%	75%
2014	$749–7113	$749–7113	$2246–21338	$1497–1871	$1497–1871	$4492–5615	$374–749	No data	$2808	$0	600%	75%

We present for the year 2014, prices paid in U.S. dollars after converting the raw South African Rand values following [28]. Shipping costs are minimal because of the use of Chinese triad shipping companies which does not require bribery payments. Poacher interview data summarized by Ken Maggs, ken.maggs@sanparks.org.

**Table 2:** Asian population estimates noted during 2010 and predicted up to 2040. From [29].

Year	PopulationEstimate/Prediction
2010	4,165,440,162
2020	4,581,523,062
2030	4,886,846,140
2040	5,080,418,644

We also collated data on rhino population variables from published [24, 25] as well as SANParks database sources (Judith Botha, judith.botha@sanparks.org). Our focus was on population estimates since 1998. We also obtained data on rhinos poached annually since 1998 from the Environmental Crime Investigation Unit of SANParks, specifically, rhino poaching data as summarized by Ken Maggs, ken.maggs@sanparks.org. We used this data to parameterize the individual-based sub-model of rhinos [30].

### Analytical approach

We consider three products associated with white rhinos traded in three largely separate markets: (1) horn for Asian consumers [16], (2) live rhinos for the South African recreational tourism and hunting market [31], and (3) the international market for reducing anxiety about rhino extinction [32]. We refer to this third market as the Species Extinction Anxiety Reduction (SEAR) market. This last market is served by private firms and Non-Government Organisations (NGOs), hereafter referred to as simply SEAR traders (see S1 Text for an explanation of this term). There are three consumer groups: horn consumers in Asia, donors to SEAR traders, and recreational hunters of rhinos. There is little overlap between these groups. Legal and illegal traders in rhino horn would engage in direct competition if consumers of rhino horn were able to choose between illegal and legal horn.

An agent-based economic sub-model [27] accounts for the international trade in rhino poaching goods [30]. An individual-based sub-model of rhinos [30] that accounts for density- and environmental-dependent birth and death processes [24] integrates with poaching predicted by the agent-based economic sub-model. Our simulations focus on outcomes within which rhinos may or may not persist over a 35-year horizon. Trends since 1998 in rhino abundance, the number of poached rhinos, and prices paid for rhino commodities allowed parameterization of these two interacting sub-models. Source code for this economic-ecological model is available at [33] along with a step-by-step guide to its use. This code is written in the Java^™^ programming language and hence is platform-independent.

**Fig 1:**
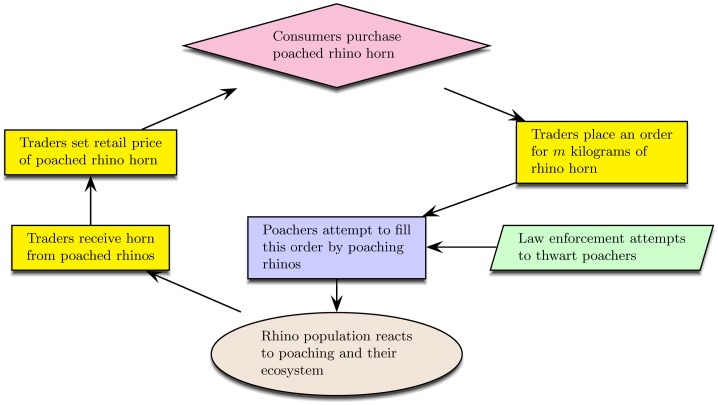
Conceptual graph of how events flow through the economic-ecological model through time.

**Fig 2:**
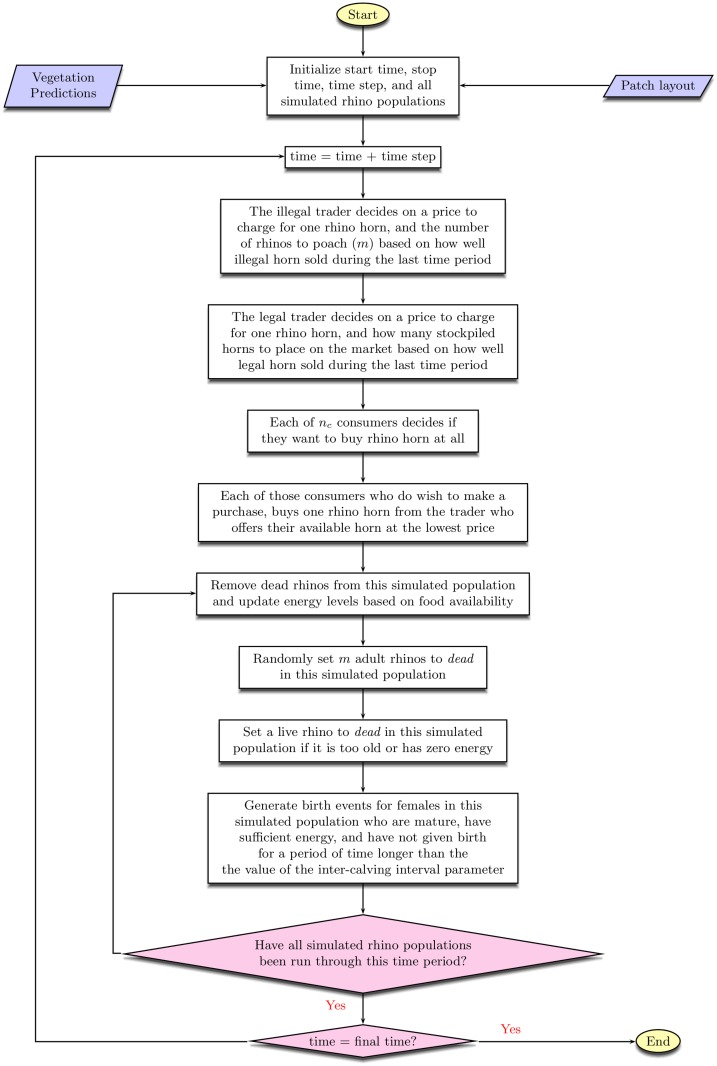
Flowchart of the economic-ecological model. How the agent-based economic sub-model interacts with the individual-based ecological sub-model is detailed.

The rhino sub-model represents the South African rhino population as they are impacted through time by their birth process, natural death process, and poaching pressure produced by the economic sub-model of the legal and illegal traders. The economic sub-model runs every 12 weeks and produces *m*, the number of rhinos to poach each week for the next 12 weeks. Then, the rhino sub-model runs every week for 12 weeks. Each week, *m* mature rhinos are randomly selected and set to the value *dead*. A conceptual, graph-based picture of how events flow through our economic-ecological model is shown in Fig 1. A detailed flowchart of the model’s operation appears in Fig 2.

We retain the distinction implied by the words *agent* and *individual* to highlight the differences between the strategic, planning-based behavior of a human versus the more instinctual, learned behavior of a foraging animal such as a rhino.

#### Economic modeling with interacting agents

In general, an agent-based economic model represents individual firms as agents and individual consumers as agents. During one step or cycle, each trader makes decisions about product re-supply and product pricing that maximizes their individual utility. Also during this cycle, each consumer makes decisions about entering a market, and once entered, purchasing decisions that maximizes their individual utility. Time is incremented, and another cycle is executed [34–36].

Building on [27], we construct an agent-based sub-model of the international trade in rhino poaching goods across three markets. Our sub-model contains a criminal network involved in rhino horn trafficking; a firm involved in seeking to trade legally in horn; the effect of a meta-firm serving the international SEAR market; and the effect of a local, South African meta-firm serving the rhino hunting market.

The effect that SEAR traders in our economic sub-model have on the poaching rate is as follows. If the number of animals that traders request to have poached is high, reduce this number to represent the effect of additional anti-poaching funds being provided by SEAR traders. The mechanism being modeled here is that as the poaching rate becomes high, donations to SEAR trader increase allowing them to spend more of their budget on aid to anti-poaching efforts. Specifically, if the number of rhinos to be poached in spite of anti-poaching measures is greater than 30 for a particular week, reduce this number by 5%. The value of 30 is intended to represent a severe poaching rate as it is about the most poaching KNP has ever experienced in one week. The value of 5% is intended to represent the small gain in anti-poaching enforcement that the injection of SEAR trader funds by themselves would have over and above existing enforcement efforts. These two values represent a conservative assessment of the effectiveness of such crisis-driven, short-term anti-poaching funding initiatives.

#### Rhino horn traders as agents

In our economic sub-model, there are two traders, one legal and one illegal. As described above, there are several levels of middlemen involved in the illegal rhino horn trade (SANParks, Ken Maggs, ken.maggs@sanparks.org). We model these as one meta-firm, *i*.*e*., we model the middlemen that directly purchase rhino horns from poachers up through the exporters as working for a single firm: the illegal trader. We argue that a criminal middleman has a restricted number of potential customers: other criminal middlemen or criminal exporters. Hence, the collection of middlemen up through the exporter acts more like a cooperative than a set of competing firms. Implicit profit sharing occurs as a middleman at one level will only be willing to purchase rhino horn from a lower level middleman if that price allows the middleman to make a profit. Ultimately, the ability of this cooperative to make a profit depends on the price demanded by poachers and the black market price that consumers are willing to pay. As long as the unit cost to this cooperative is lower than consumers’ reserve price, the illegal trader will stay in the business of rhino horn trafficking. To continue to make a profit, these illegal traders will lower or raise their black market price in response solely to the purchase decision making of consumers. Therefore, some estimate of an illegal trader’s unit cost is needed.

In our economic sub-model, the unit cost for acquiring and selling one kilogram (*kg*) of rhino horn by either trader is USD $5,000. For the illegal trader, this number is arrived at by considering that trader’s costs as follows. First, the illegal trader needs to purchase a horn from a poacher. In [37], the black market price for one kilogram of rhino horn is estimated to be between $35,000 and $60,000 with about 5% of that being used by the illegal trader to purchase the rhino horn from poachers. There is some evidence that crime syndicates typically pay small amounts for horn to people living next to parks [38] because such people, having limited economic opportunities [39], have little leverage to negotiate higher prices. Therefore, we use the lowest black market price as our estimate for what poachers are paid: $1,750 for one kilogram of rhino horn. Next, the illegal trader needs to purchase a courier’s airfare from Maputo, Mozambique to some city in Asia for $2,000. Finally, the illegal trader needs to pay the courier’s fee of $500 [40] per rhino horn or $100 per kilogram of rhino horn assuming an average pair of rhino horns weighs about 5 *kg* [41]. Using these numbers, the trader has incurred a cost of $3,850 to bring one kilogram of rhino horn to an Asian market. Thus, a conservative unit cost is $5,000.

Traders are not allowed to engage in product “dumping,” *i*.*e*., sell their rhino horns for less than their unit costs. Each week, traders always sell as many kilograms of rhino horn as there are consumers willing to purchase them. Vietnamese rhino horn merchants usually have a number of rhino horns available for inspection [42]. This implies that (a) there is no direct order placed by a customer before a rhino is poached, and (b) illegal traders maintain a buffer stock (inventory) of rhino horn.

#### Modeling syndicate supply chain dynamics

With continued anti-poaching patrols, firefights, and arrests, poaching teams have become more militarized as is evident through their carrying of AK-47s for protection against anti-poaching patrols [43] and the increased share of rhino horn revenue for the shooter (Table 1). Poaching teams have demanded that middlemen (through the transporters) pay them for each kilogram of rhino horn they deliver rather than through a flat fee for a hunt that may result in multiple rhino horns (Table 1). In this way, the Level 1 poachers realize a larger expected revenue to offset the larger expected loss (risk) they face when they set out on a hunt.

To model such supply chain dynamics, our economic sub-model represents Asian retailers, middlemen, and the aggregate of poachers and transporters. Poachers and transporters are aggregated so that a consistent price unit (a kilogram of rhino horn) can be used within the sub-model and its attendant output can be compared to observed prices (Table 1). In every cycle of the sub-model, the retailer offers USD *u*_*r*_ per kilogram for *q*_*m*_ *kg* of rhino horn and the middleman offers USD *u*_*m*_ per kilogram for *q*_*m*_ *kg* of rhino horn. In order to meet this demand for *q*_*m*_ *kg* of rhino horn, poachers attempt to poach *q*_*m*_/5 rhinos (recall that a pair of rhino horns from one rhino weighs about 5 *kg*). They succeed in poaching *q*_*p*_ rhinos in the face of anti-poaching measures directed against them. In the subsequent cycle, the retailer receives as input of 5*q*_*p*_ *kg* of rhino horn.

The authors of [44] show that firms in a multi-tier supply chain stand to make higher profits through vertical integration. Specifically for a three tier supply chain, the retailer and the supplier both increase their profits if the tasks of the middleman can be taken on by one or both, and the middleman removed (disintermediation). Because the middleman acts only to connect suppliers to retailers, the middleman adds little value to the product. Middlemen do develop suppliers in emerging markets such as rhino horn trafficking but, as the poachers gain experience, this development function ceases to be necessary.

Retailers also rely on middlemen in an emerging market to seek out suppliers. Again, once these suppliers become established, this liaison role becomes unnecessary. Theoretical justification for this tendency can be found in [45]. These authors find that once a supply chain is stable, there are economic reasons for eliminating the middleman. This result implies that before the supply chain becomes stable, *i*.*e*., in an emerging market, middlemen play a critical role in the market’s operation.

One mechanism that may gradually force the middleman out of the supply chain is through “ask and bid” price squeezing [46]. In this mechanism, poachers gradually increase their asking price for horn, while retailers gradually reduce their bid price for horn that they purchase from the middleman. The middleman accepts these new prices because the middleman is facing two monopolists who jointly completely control all of the middleman’s market share. This mechanism is represented in our economic sub-model by having within each cycle, the retailer offer an incrementally lower price to the middleman. And having the poachers in turn, place an incrementally higher price on the rhino horn that they have poached. These two actions taken together can reduce the middleman’s profit over time.

### Characterizing and estimating demand

We assume that the illegal rhino horn trade operates a standard supply chain having a non-zero stockpile or inventory. Discovery of large, private, illegal stockpiles of rhino horn in Asia might indicate that criminal syndicates are engaging in speculative rhino horn purchasing (hoarding) in anticipation of rhino extinction. The speculation is that if the rhino were to become extinct, the price for stockpiled rhino horn would be high enough for a speculator to make a profit by selling such horn post-extinction. Of course, this price would need to be high enough to allow for the lost interest from not selling the stockpile prior to the extinction event [47].

In our model, the criminal syndicate maintains two rhino horn stockpiles: one to supply rhino horn retailers, called here their *inventory*; and one for speculation purposes, called here their *speculation stockpile*. Let *D*_*T*_ be the random variable modeling demand for rhino horn in period *T*. Let *Y*_*T*_ be the random variable that denotes the amount of rhino horn placed into inventory at the beginning of period *T*. Let *Q*_*T*_ be the supply of rhino horn from poaching. Let *R*_*T*_ be the amount of this supply that is placed into the speculation stockpile. Then *Y*_*T*_ = *Q*_*T*_ − *R*_*t*_ and the hoarding rate is *R*_*T*_/*Q*_*T*_.

No evidence has been reported since 2009 that indicates criminal syndicates are building such stockpiles. Hence, we set the hoarding rate in our model to 5%. Because we have chosen a small value for this parameter, the results of our simulations, below should be viewed as conservative. In other words, if the actual hoarding rate is higher, the activities of rhino horn trafficking syndicates are having an even greater impact on rhino sustainability. Some apparent hoarding may be due to inaccurate short-term demand forecasts by illegal traders, but with our assumption of a small hoarding rate to begin with, we do not see the need to model or estimate this distinction.

Statistical estimators of trafficked horn supply, demand, and average price that are derived from modern statistical practice would be ideal, but need data before they can be implemented. We describe several such estimators in S2 Text. In lieu of such a statistical estimator, we describe the demand estimator we use in our economic sub-model that is based on United Nations estimates of population growth on the Asian continent.

#### Asian population trend estimate of demand

Asian black market retail prices for a kilogram of rhino horn (Table 3) exhibit exponential growth. If all of the rhino horns supplied from poached rhinos were sold at the prices given, and there was no customer recruitment through time, then the market demand curve based on this data would be strongly upward sloping. This is an example of the market demand curve not being a scaled version of an individual’s demand curve [48–49]. Because *Q*_*t*_ monotonically increases from 2009 to 2014, it is likely that *D*_*t*_ ≥ *Y*_*t*_ for *t* = 2009, …, 2014.

**Table 3:** Number of rhinos poached and rhino horn retail prices.

Year	Number of Rhinos Poached (*Qt*)	Retail Price (USD)per kilogram (*μt*)
2009	122	5,000
2010	333	10,000
2011	341	20,000
2012	668	65,000
2013	1004	65,000
2014	1215	97,000

The poaching numbers are from [50]. The 2009–2011 prices are from [12], the 2012 price is from [51], the 2013 price is from [52], and the 2014 price is from [53].

The author of [54] reports that there is a common practice of selling imitation (“fake”) rhino horn that is actually buffalo horn crudely shaped to look like genuine rhino horn. Up to about 90% of the “rhino horn” sold in Vietnamese shops is fake [55]. The author of [54] also reports that there are elite customers who use trusted buyers to purchase entire rhino horns for their family. There are techniques for detecting fake horn that these trusted buyers are familiar with. One conclusion that can be drawn from [54] is that the fake horn is manufactured for the low-price customers in order to hold in reserve the genuine horn for the elite customers. This anecdotal evidence suggests that the demand for rhino horn is significantly higher than the supply.

Using only this information, a quantitative estimate of demand is not possible. What can be said is that demand is higher than sales of genuine rhino horn as long as a significant amount of imitation rhino horn is sold. This implies that with the black market in rhino horn, *D*_*t*_ is almost always larger than, rather than equal to *Y*_*t*_.

Therefore, because we lack the necessary data to execute any of the sampling-based estimates of rhino horn demand (see S2 Text), we proceed instead as follows. The assumption of insatiable demand at current (illegal) production levels is represented by initializing the consumer population as follows. Create enough consumers to purchase all rhino horn poached under the maximum poaching rate of 30 rhinos per week across South Africa (20 in KNP, and 10 on private ranches). Because each pair of rhino horns weigh on average 5 *kg*, these numbers are multiplied by 5. For policies that include a legal trading scheme operating in parallel to the illegal trade, this consumer pool is doubled. Because demand for rhino horn in the near future is predicted to be about four times current sales [3], doing so is well-within current demand forecasts. The supply of legally-traded rhino horn would be sourced from stockpiles and/or shavings from live rhinos. Therefore, in the year 2014, the potential number of consumers is set to 300 (5×2×30). This value is increased in proportion to the entries in Table 2 to a maximum of 325 in the year 2033.

By a “consumer” we mean a group composed of a number of real-life individuals. The author of [56] reports an individual purchase for $2000 of rhino horn powder. At the per-kilogram prices mentioned above, this would be between 33 *g* and 57 *g* of rhino horn. Other individuals of course may purchase other amounts of rhino horn. In our model however, one of our “consumers” always buys exactly one kilogram of rhino horn at each purchase event. Hence, one of our “consumers” represents approximately 18 to 30 real-life individuals. By doing so, we ignore the variability in the amount of purchased rhino horn and in-effect, lump approximately 18 to 30 real-life purchase events into one purchase event. Hence, our sub-model's purchase event time series should be viewed as the aggregate behavior of groups of approximately 18 to 30 real-life individuals.

Next, consumer behaviors start with the decision to enter the rhino horn market or not. If there is a media campaign aimed at potential rhino horn consumers that delivers a message that rhino horn has no medicinal value, some of the potential consumers may decide to not try to purchase rhino horn. This media campaign effect is represented as follows. Let *n*_*pc*_ be the number of potential consumers each week. Let *p*_*m*_ be the effectiveness of a horn-is-not-medicine media campaign run in the country where the consumers live. If *p*_*m*_ is close to 1.0, the chance that a randomly chosen potential consumer will decide to buy rhino horn is close to zero. Let *N*_*c*_ be binomially distributed where there are *n*_*pc*_ trials, and the success probability is 1 − *p*_*m*_. Sample once from this distribution to find *n*_*c*_, the number of consumers for that week who enter the market for rhino horn.

To represent the absence of demand reduction campaigns, *p*_*m*_ is set to 0.0. Because there is little evidence that these campaigns are effective [57], we set *p*_*m*_ to 0.15 for any policy that includes a demand reduction campaign component.

Now, rhino horn purchases may be simulated. Each consumer buys one kilogram of rhino horn from the trader offering it at the lowest price as long as this price is below the consumer's reserve price of USD $60,000 [58]. The number of kilograms of rhino horns the illegal trader sells each week need not equal five times the number of rhino horn pairs poached the previous week. This is because it is assumed that the illegal trader maintains a buffer stock of rhino horn. See [30] for a detailed, step-by-step description of the agent-based economic sub-model’s operation.

#### Estimating intra-syndicate prices

Information on the price of rhino horn paid between members of different levels of the criminal syndicate (Table 1) is used to estimate a lower bound on the criminal syndicate’s unit cost. As the raw data is in South African Rand, we used the average “bid” exchange rates (Table 4) to convert to US Dollars [28]. Doing so appears to be reasonable because an Asian retailer would convert their currency, say USD to Rand in order to purchase the rhino horn from the port exporter.

**Table 4:** Average “bid,” and “ask” exchange rates USD to ZAR. Extracted from [28].

Year	bid (ZAR/USD)	ask (ZAR/USD)	1/bid
2006	6.75050	6.78812	0.14814
2007	7.03077	7.06926	0.14223
2008	8.23218	8.27505	0.12147
2009	8.40084	8.44468	0.11904
2010	7.30053	7.34412	0.13698
2011	7.23125	7.27131	0.13829
2012	8.19734	8.22357	0.12199
2013	9.63485	9.65134	0.10379
2014	10.68529	10.70243	0.09359

The 2014 averages are for the first six months of the year.

The sets of intra-syndicate price observations are used to estimate a lower bound on the criminal syndicate’s unit cost. We focus on the Asian retailer’s unit cost. This cost is equal to the price charged by port exporters for a kilogram of rhino horn. Port exporters in-turn, deliver this rhino horn by meeting the middleman’s price and paying for shipping. Hence, a lower bound for the Asian retailer’s unit cost is the sum of the price charged by transporters to middlemen plus any shipping costs. The author of [59] reports that a portion of the rhino horn shipped to Vietnam is re-shipped to China. This suggests that the Chinese market for rhino horn is large and stable. We thus predict that if criminal syndicates are forced to cut costs in the face of a legal trade competitor, they will at minimum reduce their shipping costs. And the main way they will do that is by switching all Vietnamese shipments to the existing shipping method used to transport rhino horn to China: ocean transport. Hence, lower bounds for the Asian retailer’s unit costs are the prices paid by middlemen to transporters. This lower bound unrealistically leaves the middleman making a zero profit. The actual, positive profit of middlemen can be computed once prices paid by retailers to middlemen are known.

### Parameter values to represent effectiveness of anti-poaching measures

The parameter *p*_*a*_ represents how effective anti-poaching patrols are at curbing poaching. In the model, *p*_*a*_ is the probability that a poaching expedition will be interdicted before it has been able to kill any rhinos. To represent current effectiveness, this parameter is (optimistically) set to 10%, *i*.*e*., there is a 10% chance that a poaching expedition will be thwarted from poaching rhino. This value is arrived at as follows.

There were 174 poachers arrested in 2014, 36 of which were deceased. There were 827 rhinos poached in 2014 in KNP. An estimated 4329 poachers entered KNP typically in gangs of three (some of these are the same poachers entering more than once). There were 111 contacts with rangers and 80 sightings of poachers by rangers. That translates to 573 poachers interacted with or seen by rangers. That in-turn, translates to a 4% chance of being arrested. There is a 0.8% chance of being killed. These arrest rates have been relatively stable across previous years even though the number of poachers entering the park has increased by 32% from 2013 to 2014 because the number arrests have also increased by 32% (Ken Maggs, ken.maggs@sanparks.org). For purposes of this analysis then, a very optimistic value of 10% (over twice the apparent success rate of 4%) is used for all non-disruptive strategic response scenarios other than Pro-active Protection. For that strategic response scenario, *p*_*a*_ = 0.20 to represent a doubling of the (very optimistic) estimate of the effectiveness of anti-poaching efforts that is consistent with the definition of this strategic response scenario. Recall that the Pro-active Protection scenario involves greater support for anti-poaching patrols, but without the integrated law enforcement effort and development of legal economic opportunities for people living next to parks. These latter two initiatives are needed to curb the operations of the criminal networks that sponsor the poaching expeditions because they reduce the ability of these networks to recruit, organize, and fund poaching expeditions in the first place. Scenarios that combine criminal network disruption with the creation of economic opportunities for people living next to parks therefore, use *p*_*a*_ = 0.60 to represent these significantly more effective anti-poaching efforts.

### Rhino individual-based model

An individual-based sub-model for the South African rhino meta-population is developed along the lines of the prairie vole (*Microtus ochrogaster*) individual-based model of [60]. As with the model of [60], the rhino sub-model is stochastic in that one run over a particular time period may not produce the same history of abundance and dispersal as another run over the same period. Therefore, many replications of the sub-model over the same time period are needed so that at each time point, both the expected value of abundance and extinction probability can be computed.

#### Rainfall predictions and available vegetation

**Fig 3:**
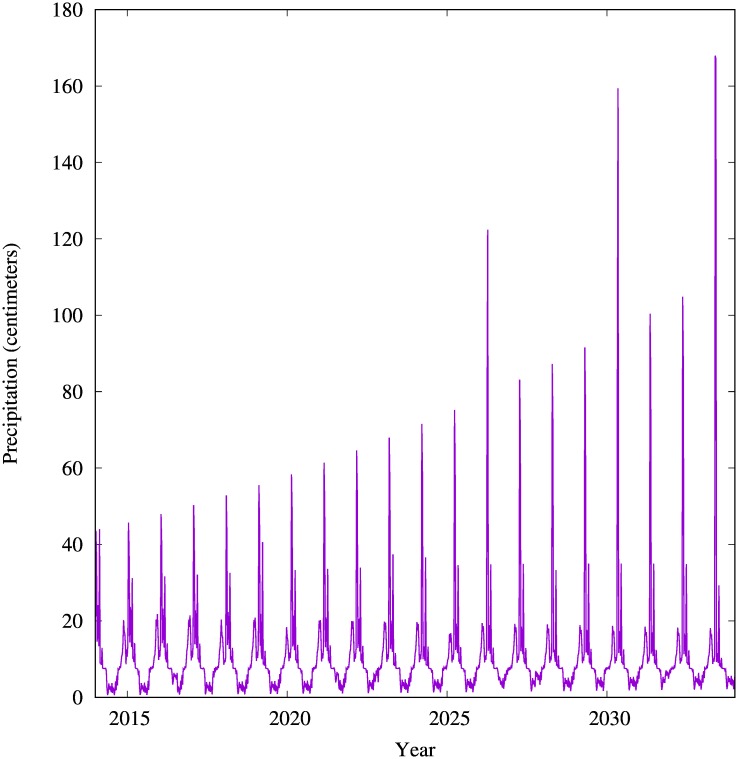
Predicted average rainfall in KNP per week (centimeters). Predictions are computed using a quasi-periodic rainfall model [30] fitted to KNP rainfall data from 1903 to 2014. Average rainfall is a proxy for new vegetation.

As described in [30], rainfall predictions for KNP over the simulation’s time interval (Fig 3) are found by evaluating a mathematical model that has been statistically fitted to rainfall observations. Predicted rainfall is used as a scaled proxy of available vegetation.

#### Operation

We model the age, gender, energetic budget, location, and status of each individual rhino living and dying in enclosed patches. By doing so, we also model interactions of these rhinos with their environment—namely seasonal fluctuations in food availability; and interactions with each other through the effects of their spatial density on their birth and mortality rates [30].

#### Parameter values

We use biologically realistic population dynamics parameters and their values in the simulations (Table 5). There are two sub-regions (KNP and ranches) each with four patches. The initial age distribution is Gaussian with a mean of 7.5 years, and a standard deviation of 3 years truncated between one week old and the life expectancy of a rhino which here, is 38 years [61] (see Table 5).

**Table 5:** ABM and IBM parameters along with their reference values and economically/ecologically valid intervals used in the sensitivity analysis.

Name	Notation	Units	Value (valid interval of values)	Source of Value
Trader Learning Rate	*ra*	unit interval	0.4 (0.2, 0.6)	[27]
Consumer Reserve Price	*reserveprice*	U.S. dollars	60000 (50000, 70000)	[37]
Legal Trader Maximum Capacity	*maxcap1*	number of horns	35 (30, 40)	[3]
Illegal Trader Maximum Capacity	*maxcap2*	number of horns	35 (30, 40)	[3]
Average Weekly Food Intake	*wfi*	kg	140 (120, 160)	[62]
Life Expectancy	*le*	years	38 (32, 42)	[63]
Maturation Age	*ma*	years	4 (3.5, 4.5)	[64]
Maximum Energetic Budget	*meb*	weeks	5 (4.5, 5.5)	after [60]
Mean Energetic Budget	*meaneb*	weeks	4 (3.5, 4.5)	after [60]
Juvenile Energetic Budget	*jeb*	weeks	3 (2.5, 3.5)	after [60]
Intercalving Interval	*intercalv*	years	2.5 (2.5, 5)	[64]
Available Vegetation	*av*(*t*)	g/m2	(as given in Fig 3)	see **Rhino individual model** section

#### Simulating future scenarios

**Fig 4:**
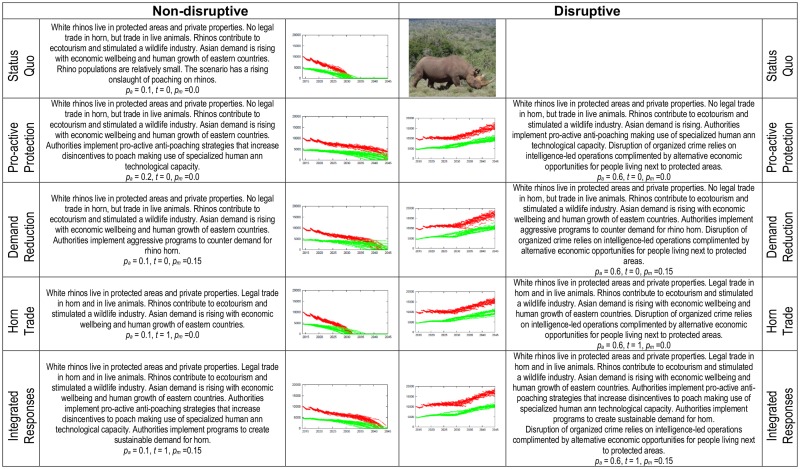
Summary of strategic response scenarios and consequences for white rhino populations. KNP rhinos are in red, and rhinos in South African private ownership are in green. Predictions are computed with and without strategies aimed at disrupting rhino horn trafficking syndicates. The parameter *p*_*a*_ is the effectiveness of anti-poaching efforts and *p*_*m*_ is the effectiveness of demand-reduction marketing campaigns on a scale of 0 to 1. The parameter *t* reflects legal horn trade (1) or not (0). During 2012 an estimated 10641 white rhinos lived in National Parks, 3710 in Provincial Reserves and 4527 in Private Reserves [17].

Policies combating rhino horn trafficking seek to avoid non-sustainable solutions that accelerate poaching-led extinction risks. We first checked what the outcomes would be for the current status quo. Four additional strategic scenarios required evaluation—intensified protection, improved demand reduction, legal horn trade, or a scenario that integrates all three of these strategic responses. For each of these scenarios we started from the present status quo and used our economic-ecological model to evaluate outcomes over a 35 year horizon (Fig 4). We altered the effectiveness of anti-poaching efforts (*p*_*a*_) and the effectiveness of demand-reduction marketing campaigns (*p*_*m*_) on a scale of 0 to 1, while allowing *t* to reflect legal horn trade (1) or not (0).

We re-ran the above scenarios, but applied a disruptive policy to our model. Our disruptive policy is two-phased. The first phase consists of smart analyses to allow creation of actionable intelligence [65] that informs criminal and civil legal responses [66]. The second phase is the creation of alternative legal economic opportunities for people living next to parks. These opportunities in-part consist of the sharing of benefits accrued from protected areas with people alienated by traditional conservation philosophy (see [5]). And, such opportunities should help to combat the exploitation of disenfranchised people by rhino horn trafficking syndicates (see [67]). Disruptive interventions have consequences similar to an aggressive increase in the effectiveness of anti-poaching. Again, we evaluated outcomes over a 35 year horizon.

## Results

### Model validation

**Fig 5:**
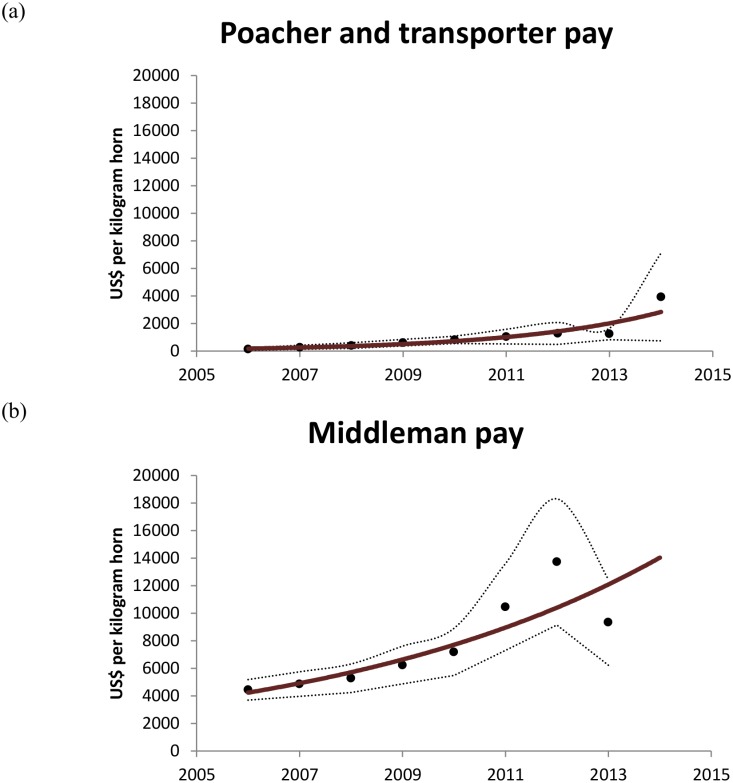
“Bid” and “ask” prices for illegally traded rhino horn. Model output (solid lines) of the price that poachers ask middlemen to pay for a kilogram of rhino horn (a), and the price that Asian traders bid to buy a kilogram of rhino horn from middlemen (b). Symbols are the observed prices paid with the broken lines representing the range of prices paid.

The economic sub-model predicts prices that Asian traders pay to middlemen that are close to the prices observed since 2006 (Fig 5). Predicted prices range from ≈$4500 to ≈$14000 per kilogram horn and increase exponentially from 2006 to 2013. Observed prices range from $3703 to $18299 per kilogram horn. Predicted prices paid by middlemen to poachers and transporters range from $60 to $2200 per kilogram horn, overlapping with the observed price ranges of $59 to $7112. Our economic sub-model thus realistically predicts the changing price dynamics in the illegal supply chain from 2006 to 2013.

The economic sub-model also predicts the number of poached rhinos which serves as input for the rhino sub-model. The resulting output from the rhino sub-model predicts population sizes that are within the 95% confidence intervals of observed estimates for 9 of the 13 years of available rhino population estimates (Table 6).

**Table 6:** Comparison of rhino population estimates derived from the economic-ecological model with estimates obtained through aerial surveys. See [23].

Year	Observed population estimates	Predicted population estimates
1998	2674 (1987–4047)	2706
1999	2938 (2295–4043)	3090
2000	2683 (1965–3629)	3401
2001	4552 (3320–6673)	3764
2002	4223 (3604–5466)	4217
2003	4765 (3710–5627)	4841
2004	5308 (3631–6434)	5465
2005	6974 (6026–8022)	5990
2006	8893 (7286–10902)	6704
2007	9119 (7665–12438)	7677
2008	11498 (9734–15619)	8601
2009	No survey	
2010	10466 (9320–11360)	10929
2011	No survey	
2012	10495 (8510–12492)	8453

We provide the 95% confidence intervals for observed population estimates. Rhino population data maintained by Judith Botha, judith.botha@sanparks.org.

Mean absolute percentage error (MAPE) computations indicate that our rhino abundance model is on average 12.2% different from actual abundance. See S3 Text for the definition of this cross-validation statistic. This amount of error lends some validity to the modeling of rhino abundance interacting with an economic sub-model. Our economic-ecological model is thus a robust reflection of the economic and ecological dynamics associated with rhinos given that it tracks price changes paid to poachers as well as rhino population changes in KNP.

Consider our Disruptive, Integrated Response scenario illustrated in Fig 4. This scenario consists of authorities pursuing aggressive disruption of trafficking syndicates while providing economic opportunities for people living next to parks. The main conclusion of this article is that under this scenario, the South African rhino population is sustainable. The sensitivity analysis reported in S4 Text indicates that this conclusion is not unduly affected by possible parameter misspecification.

### Scenario predictions

Intensified anti-poaching, demand reduction, and competitive horn trade policies result in non-sustainable solutions (Fig 4). Under the status quo, extinction probability increases rapidly from 2027 onwards. Purist intensified protection and demand reduction policies result in extinction risk rapidly increasing by 2037 –a 10 year extension of rhino existence. A purist trade policy will only buy one extra year—extinction risks increase rapidly from 2028 onwards. Combining these three policies still results in rapid extinction risk increase by 2028. But what happens if each of these policies is complemented with disruptive interventions that target organized crime syndicates while at the same time providing economic opportunities for people living next to parks? In those scenarios, sustainable solutions are obtained irrespective of the policy implemented (Fig 4).

## Discussion

Curbing wildlife trafficking has become a key international focus [68]. Authorities have several approaches at their disposal to address the illegal trade in wildlife products [8]. For rhinos, most policies that have been debated combine increased protection with either demand reduction as championed by anti-trade proponents [9–11]–or legal supply options championed by pro-trade activists [12–14]. Our simulations suggest that protecting rhinos *in-situ*, reducing demand, and providing legal supply as policies to curb threats of rhino horn trafficking all result in ultimate rhino extinction. The author of [69] illustrated that most anti-poaching methods failed to protect rhinos. Our analysis also shows that demand reduction and legal supply policies carry failure risks. Therefore, policies need complimentary responses. Trafficking syndicate disruption across the complete illegal supply chain coupled with increases in legal economic choices that improves peoples’ quality of living may assist authorities to achieve sustainable outcomes.

Present interventions have not curbed the rhino poaching onslaught. Poaching rates continue to rise since 2006 [17] and key rhino populations may decline in the future [25]. At present, rhino management policies allow for no legal horn trade [70], but permit live African rhino trade and legal sport hunting [31]. In addition, rhinos have high ecotourism value [71] and stimulate a vibrant wildlife industry [31,72]. Rising Asian demand for horn is associated with economic well-being of eastern countries [73]. Present rhino populations are relatively small [17] and threatened by the rising onslaught of poaching. This present scenario and associated dynamics predicts continued decline in rhino population size with rapid increases in extinction risks of rhinos by anytime from 2020 to 2050 [30].

The existence of multiple solutions in the dynamics of rhino horn trade introduces uncertainties that fuel trade versus no-trade debates [48]. Agencies that inform international agreements such as CITES, suspect multiple solutions and evoke precaution [74] when they believe there is a chance that reality will settle into a non-sustainable solution. But continuation of the status quo as a result of political and bureaucratic inertia also risks a non-sustainable solution. A consequence is that various policy options arise [8], but assessment of the outcomes of these is relatively rare. For example, the African Rhino Action Plan [75] inherently evaluates extinction risks and then proposes several actions. But the potential for these actions to achieve stated objectives is not explicitly assessed. When conducted, expert-based risk analyses indicate that benefits consistently exceed risks for those policies that in some or other format legally match supply and demand [76]. These analyses do, however, contain high levels of uncertainty [76]. And, they are premised on the two assumptions that (a) the scale and drivers of demand are known and can be managed; and (b) demand will redirect itself towards legally traded horn only. In addition, most proposed legal trade models (*e*.*g*. [13]) assume relative equilibrium economic dynamics [77] with near perfectly understood markets. The trade, illegal or legal, in wildlife goods like rhino horn has many features that challenge the assumptions of such simple economic models [48].

Our study has attempted to overcome some of these uncertainties. Individual-based ecological [60] and agent-based economic [27] modeling can track the trends in prices paid to poachers at various levels of the supply chain, but more importantly can track rhino population changes. Predictions are thus based on approaches that track historical patterns, thus reducing some of the uncertainty surrounding the prediction that introducing legal trade in rhino horn to the current status quo may lead to non-sustainable rhino populations. This is also the outcome of introducing more aggressive rhino protection and demand reduction campaigns. Our results have some parallels with a recent study examining the socioeconomic drivers of poaching, based on data collected from 1990 to 2013 [14]. Increases in anti-poaching enforcement and penalties for violators can maintain rhinos, but may carry high financial costs. Our model predicts sustainable rhino populations only when intensified protection, demand reduction, or legal trade in rhino horn are complimented with trafficking syndicate disruption combined with creation of alternative economic options for people living next to parks. This prediction challenges the current global support for the increased development of militaristically-oriented anti-poaching tactics (see [67, 78] for critiques of this militaristically focused approach to biodiversity protection). Our prediction is also at odds with proposals to increase sanctions [79] that ultimately result in further alienation of stakeholders living next to protected areas [43]; or potentially, across a nation [80].

Our findings may appear to contradict that of [14] who conclude increased protection funded through rhino horn sales revenue can save rhinos. Increased protection would require crime disruption—similar to our analyses. In addition, lasting outcomes of reduced crime is more likely in environments that provide equal rights in fair benefit sharing [81]. The authors of [14] imply the same key requirements as what our analyses highlight. It is the funding of these initiatives that dichotomize the debate. Given that rhino horns are part of a suite of commodities that organized crime targets [16] authorities that deal with organized crime will also deal with rhino horn trafficking. Safe and secure environments free of organized crime of any sort as well as social development directed at improving people’s well-being depends on good governance [7]. It is unrealistic to expect the provision of good governance to depend on revenue generated by the use of a natural resource like rhino horn.

Our findings also contradict the opinions of authors who advocate the sale of rhino horn as a way to curb rhino poaching through a mechanism using a Central Selling Organization (e.g. [13]). We do not explicitly model the mechanism of trade—we simply ask whether trade, in whatever format, will have consequences for rhinos. Even so, a key challenge for any mechanism of legal trade aimed at curbing rhino horn trafficking would be the ability to undercut the prices offered by illegal traders. Criminal networks are able to engage in predatory pricing (undercutting) with legal traders because criminal networks have less overhead and can access capital without the need for borrowing [82]. Note that a government-run central selling organization would need to borrow money through bonds if its operation was more expensive than its tax receipt revenues—identical to any legal, private trader. Criminals undercut legal traders for a range of commodities including gambling services [83], cigarettes [84], Pacific crab harvesting [85–86] and the international timber trade [87]. An extreme form of such criminal predation is the take-over of all trade in a commodity by a criminal organization via their practice of infiltrating a legal business and eventually taking control [88]. These examples challenge the potential of trade mechanisms being able to force criminal syndicates to operate at a loss. Our results clearly illustrate that disrupting organized crime is a key element disregarding whether authorities allow trade in rhino horn or not, as well as whatever mechanism authorities use if trade is allowed.

We do not know of a documented case of a legal trade mechanism driving out a black market without the assistance of strong and effective law enforcement. Indeed, black markets appear to be very resilient in the face of legal competition. In addition to the cases cited above, one can look at the recent record of marijuana legalization in some states in the United States. The author of [89] delineates how legalization has failed to shutdown illegal marijuana sales. Specifically, allowing legal trade can indeed lower the product’s retail price, but often not enough to cause traffickers to view their participation as unprofitable. For example, several years into legal marijuana in some states, 2.2 million U.S. dollars of illicit marijuana was seized at a United States border crossing [90]. In this case, illicit marijuana continues to be produced in large quantities in the face of a lower profit margin for its smugglers. Tobacco is another case of an illicit market co-existing with a legal one. The authors of [91] estimate that 21% of all tobacco sales in the United States is illicit.

A well-known example of legal trade apparently driving illegal traders out of a market is the 1933 repeal of Prohibition in the United States. But the actual mechanism involved in this case only serves to reinforce the importance of disrupting criminal syndicates. As a case in point, just after Prohibition was repealed, the Governor of the state of Washington in the United States assigned Admiral Gregory the task of shutting down the illicit alcohol trade in that state [89]. Gregory's successful strategy consisted of the following five steps.

1. Make the entry cost for a legal trader very low and impose very low taxes initially.
2. Allow illegal traders to voluntarily switch to being legal traders without any legal penalties as long as they agree to pay a tax on each liquor sale.
3. Allow legal liquor producers many legal outlets for their product so that they can use their economy of scale production system to set a low selling price but still turn a profit.
4. Have rigid, effective law enforcement: successfully detect any illegal trader and wield the necessary legal authority to permanently shut down that trader's business.
5. Raise the tax only after all illegal traders have left the market.

The present illicit trade in rhino horn does not appear to share three key aspects of this strategy. First, wildlife traffickers are typically criminal syndicates engaged in a variety of illegal activities. To simply declare them to be legal without any criminal prosecution of their other business activities would be illegal under South African law. Second, the world's record of successfully shutting down illegal traders has clearly been a failure. Third, given that two-thirds of South African rhinos live on reserves, it seems unlikely that private rhino owners would be able to achieve enough trade volume as to make illegal trade unprofitable. This is because traffickers, with their relatively lower overhead costs would almost always be able to find a profit-making price that is as low as any private owner’s profit-making price. For private owners, the only way to set a lower price that is still profitable would be through high-volume production, i.e., much larger numbers of privately-owned rhinos. These policies carry risks to sustainability because their implementation would incentivize intensive breeding of rhinos, a practice that has been shown to carry significant challenges for other species [92–93].

The authors of [94] provide evidence to support their conclusion that the legal sale in elephant ivory from 1989 through the one-time sale of ivory stocks in 2008 likely produced the recent, catastrophic rise in poaching. This is the opposite of what would be expected if the phenomenon was governed by classical (static) economics. These authors suggest that several factors could be operating. These are (a) ineffectual disruption of ivory trafficking by law enforcement, (b) fencing opportunities created by legal traders, (c) market expansion due to the removal of the stigma surrounding ivory consumption, and (d) perceptions by poachers that the ivory market is growing. This last factor may account for the permanent 65% increase in elephant poaching that occurred in spite of an observed drop in ivory prices.

But could legal trade in ivory be sustainable? The authors of [95] employ an elephant population dynamics model coupled to data-based forecasts of ivory demand to address this question. Their modeling results suggest that demand is much too great to supply it with a sustainable elephant harvesting strategy. In other words, lifting the ivory trade ban would lead to the elephant’s extinction.

We emphasize that disruptive policies is not simply increasing traditional anti-poaching effort. In our model, the parameter *p*_*a*_ set at the value of 10%, represents an optimistic poacher interception rate by anti-poaching units who focus their efforts on poachers in the first tier of the criminal syndicate involved. At present, the realized interception rate within KNP is only 4% (Ken Maggs, ken.maggs@sanparks.org). When we set *p*_*a*_ to 60%, a 15-fold increase in interception success, we are modeling a qualitatively different approach to achieve such a level of effectiveness at curbing rhino poaching. If within-reserve poacher interception linearly relates to funds spent on anti-poaching, then budgets have to increase 15 times, a financially challenging way to increase anti-poaching effectiveness enough to achieve sustainability (see [14]). Such an increase in effectiveness can only be realistically achieved through the enactment of two parallel initiatives. First, disruption of the entire syndicate's operations from the poacher on the ground up through the four levels of middlemen. Second, a reduction in the economic need to poach rhinos by people living next to parks through the development of alternative economic opportunities for them.

How may such criminal network disruption be achieved? Crime networks engaged in wildlife trafficking span beyond those protected areas [16] that are strongholds of species of interest such as African rhinos. Crime networks also extend over several countries beyond the jurisdiction of any one law enforcement authority. Federated databases can overcome disjoint information kept in different databases, while social network analyses can provide law enforcers with targeted responses that maximally disrupt a criminal network [20]. In our model, setting *p*_*a*_ to 60% represents the end-effect of such criminal network disruption.

The entrance of trans-national organized crime syndicates into rhino horn trafficking [16] has produced challenges that wildlife protectors have never faced before. Our simulations illustrate that traditional policies [8], fueling lengthy debates in some instances [13, 48], is not enough to curb rhino horn trafficking [14, 67]. Innovative collapse of trans-national crime syndicates is a prerequisite disruptive policy that, in association with establishing alternative economies for people living next to parks may substantially reduce the risks of rhino extinction.

## Conclusions

The present debate about whether to allow legal trade in rhino horn gives low importance to two critical aspects of the rhino slaughter crisis. These are the efficient criminal networks that fund the slaughter; and the attractiveness to people with no legal economic alternatives to participate in such criminal operations. The debate needs to change to include multi-faceted strategies that contest the slaughter across all of its drivers and at all of its levels. These strategies ultimately depend on good robust governance [7], which, when implemented, could create several options to use natural resources sustainably. We have shown through our data-validated modeling results how such strategies can be evaluated against their likelihood of avoiding the extinction of the rhino. Hence, we recommend that a model such as ours be used to assess the potential success of any policy that is intended to promote survival of the rhino. More generally, our modeling and evaluation approach could be applied to any species for which anthropogenic pressures are putting its survival at risk.

## Supporting Information

S1 Text Definition and justification for the term *SEAR trader*. (DOCX)

S2 Text Statistical estimators that could be used to estimate rhino horn demand, supply, and retail price. (DOCX)

S3 Text Cross-validation statistics used to assess the economic-ecological model’s validity. (DOCX)

S4 Text Sensitivity analysis of the economic-ecological model. (DOCX)
