# Supplementary material for: Combating Rhino Horn Trafficking: The Need to Disrupt Criminal Networks
Source: PLoS One. 2016 Nov 21;11(11):e0167040. doi: 10.1371/journal.pone.0167040 (PMC5117767; doi:10.1371/journal.pone.0167040)
Supplement: S1 Text — (DOCX) [file pone.0167040.s001.docx]

**S1 Text**

**SEAR traders**

Labeling well-meaning conservation NGOs “anxiety reduction traders” may appear to be overly cynical hence we justify this terminology as follows. The authors of [1] find that a random sample of Chilean citizens are willing to pay (WTP) for the conservation of an endemic moss having no economic value because for them, the moss has “ethically motivated existence value.” In other words, these individuals believe that it would be ethically wrong for this moss to become extinct -- and they are willing to pay some amount of money to see that it won't. Such concern for species extinction is not limited to Chile. A 2011 Gallup poll estimates 60% of Americans worry about plant and animal extinctions [2]. Conservation NGOs however, are in a challenging enterprise: they have well-grounded conservation goals but need to find a way to finance their conservation projects mainly through donations from individuals from all walks of life who have a range of knowledge and understanding of the true state of the planet’s biodiversity. Further, such potential donors will not receive anything of tradeable value in return for their donation. In answer this challenge, conservation NGOs have few options available to them. One such option is a particularly structured marketing campaign.

For example, the World Wildlife Fund (WWF) has about 5 million members world-wide with about 1.2 million of those in the United States [3]. The WWF's marketing strategy consists of two main steps. These are as follows.

1. Identify potential donors as those already holding “ethically motivated existence values” in the sense of [1] for species that are at risk of extinction. Do this by either acquiring targeted mailing lists or by identifying media outlets (including social network sites) that cater to such individuals.

2. Using these outlets, deliver an advertising message that consists of first, a message intended to generate fear or anxiety about the possibility that a particular animal might become extinct, e.g. tigers, polar bears, seals, elephants, rhinos, or monkeys. Then, just below this message offer a way to fend off this feared event by donating to the WWF. See for example, [4].

See [3,5] for detailed examples of such WWF advertisements. See [6] for a general discussion of this marketing strategy. The WWF therefore, is advertising to pre-disposed individuals a remedy for their anxiety surrounding potential species extinctions, namely donating to the WWF. In-effect then, the WWF is in the business of anxiety reduction, *i.e.,* it is an anxiety reduction trader. We assume this is the business model used by many biodiversity-focused NGOs. Inadvertently, SEAR traders benefit if public perception prevails that the rhino is headed for extinction due to poaching. In other words, if rhinos cease to be endangered, the global feeling of anxiety towards the future of rhinos would be reduced, thus reducing the demand for the service SEAR traders are selling: anxiety reduction.

Note that in this discussion, the actual abundance of a species and its actual extinction risk are replaced by whatever media-generated sense of these two values that are believed by potential SEAR trader customers. It is assumed herein, however, that SEAR traders have a fairly realistic sense of a particular species’ extinction risk and use that knowledge to guide their decisions concerning which anti-poaching projects to financially support.

**References**

1. Cerda C, Barkmann J, Marggraf R. Application of choice experiments to quantify the existence of an endemic moss: A case study in Chile. Environment and Development Economics. 2012; 18: 207-224.

2. Saad L. Water issues worry Americans most, global warming least. In: Newport F, Lanham F, editors. The Gallup Poll: Public Opinion 2011. MD: Rowman and Littlefield Publishers, Inc.; 2013. p. 109.

3. Flanagan E. Visual persuasion and communicator credibility in WWF environmental advertising campaigns. School of Communication Studies, James Madison University. 2008. Available: <http://ellieflanagan.weebly.com/uploads/1/5/2/8/15287628/persuasionpaper.docx>. Accessed 17 October 2015.

4. WWF 2015. Threats. Available: <http://www.worldwildlife.org/species/rhino>. Accessed 17 October 2015.

5. Henneberry R. How to sell using fear. 2013. <http://blog.crazyegg.com/2013/01/08/how-to-sell-using-fear/>. Accessed October 17, 2015.

6. Adler T. Environmental advantage: Marketing the messages. Environmental Health Perspectives. 2002; A538-A585.
