# Supplementary material for: Combating Rhino Horn Trafficking: The Need to Disrupt Criminal Networks
Source: PLoS One. 2016 Nov 21;11(11):e0167040. doi: 10.1371/journal.pone.0167040 (PMC5117767; doi:10.1371/journal.pone.0167040)
Supplement: S2 Text — (DOCX) [file pone.0167040.s002.docx]

**S2 Text**

**Statistical estimate of trafficked horn supply based on customs seizures **

One way to estimate the amount of horn being trafficked ($Q_{t}$) is to use data on the number and sizes of rhino horn seizures by customs oﬃcials. Let $\gamma$ be the interception (seizure) rate: the portion of $Q_{t}$ that is intercepted by customs officials. Let $I_{t}$ be the amount of rhino horn in kilograms that is seized in period $t$. Then $\hat{Q}_{t}=I_{t}/\gamma$. And under the above assumption concerning speculative purchasing of rhino horn, $\hat{Y}_{t}=0.95\hat{Q}_{t}$. A way to estimate $\gamma$ is needed before seizures data can be used to compute $\hat{Q}_{t}$. The authors of [1] develop one way to estimate $\gamma$ for trafficked ivory that involves the modeling of the effectiveness of law enforcement within different countries via a Bayesian hierarchical latent variable statistical model. A similar model could be developed for trafficked rhino horn.

**Statistical estimate of demand**

If there is unmet demand ($d_{T}>y_{T}$), then sales should be viewed as a censored observation of demand. The cumulative distribution function (CDF) estimator developed in [2] for demand takes this into account. One could use this CDF to update the number of consumers each period by sampling once from an updated, discrete demand CDF. This update would be accomplished by computing an estimate of a nonparametric demand distribution based on all observed sales data from 2009 up to that period's time point, $T$. Then, demand through time could be estimated from observations of actual sales of rhino horn in Asia. Let $Z_{t}=\min\{D_{t}, Y_{t}\}$ and $F_{T}(d)$ be the CDF of $D_{t}$ evaluated at $d$. Denote the order statistics of $Z_{1},\ldots,Z_{T}$ with $Z_{(1)},\ldots,Z_{(T)}$, *i.e.,* $Z_{(i)}\leq Z_{(j)}$ for $i<j$. For $d\leq Z_{(T)}$, the Kaplan-Meier estimate of $F_{T}(d)$is

$$\hat{F}_{T}(d)=1-\prod_{t:Z_{(t)}\leq d} \left(\frac{T-t}{T-t+1}\right)^{\delta_{(t)}}$$

where $t=1,\ldots,T$, and $\delta_{t}=I_{\{D_{t}\leq Y_{t}\}}(Y_{t})$.

Note that $Z_{t}$ equals $Y_{t}$ if the entire inventory is sold during this period, (called a “stock out”'), or equals $D_{t}$ if not all of $Y_{t}$ is sold this period. In this latter case, $D_{t}$ equals the portion of $Y_{t}$ sold during the period. If $D_{t}>Y_{t}$ for all $t$ because customers are either turned away from shops having sold out their rhino horn inventory or are offered only imitation rhino horn [3], then $\delta_{(t)}=0$ for all $t$. In this case, $\hat{F}_{T}(d)=1-1^{T}=0$ for any values of $d$ such that $d\leq Z_{(T)}$ and hence, $P(D_{T}>d)=1$. A realization would be drawn from this distribution by computing the value $d^{*}=\inf\{d: r^{*}\leq\hat{F}_{T}(d)\}$. Note that total sales of trafficked rhino horn from 2009 to the present are needed to implement this demand estimator.

**Statistical estimate of what consumers pay on average for trafficked horn**

Let $X_{j}$ be the amount (in kilograms) of rhino horn purchased by the $j^{\text{th}}$ Asian consumer, and $U_{j}$ be the price it sold for.

Let $\tau_{X}=\sum_{j=1}^{M} X_{j}$,

$\tau_{U}=\sum_{j=1}^{M} U_{j}$,

and $\mu=\tau_{U}/\tau_{X}$.

The value of $\tau_{X}$ is the total weight of all purchased rhino horn, and $\mu$ is the average per-kilogram selling price.

A cluster sample can be used to estimate these quantities when $M$ is unknown. First, let $N$ be the number of clusters partitioning the population of Asian rhino horn consumers. Say that a size-$n$ sample of these clusters is taken. For $i=1,\ldots,n$, let $x_{i}^{(c)}$ be the total weight of purchased rhino horn in cluster $i$, and $u_{i}^{(c)}$ be the total revenue from these purchases. Then,

$$\hat{\tau}_{X}=\frac{N}{n}\sum_{i=1}^{n} x_{i}^{(c)}, \hat{\tau}_{U}=\frac{N}{n}\sum_{i=1}^{n} u_{i}^{(c)},$$

and

$$\hat{\mu}=\frac{\widehat{\tau_{U}}}{\widehat{\tau_{X}}}$$

[4].

Define the $i^{\text{th}}$ cluster to be a street of traditional medicine shops in a particular Asian city, or an online storefront that offers rhino horn for sale. There are $m_{i}$ rhino horn consumers in this cluster. Say that rhino horn can be purchased every day from 9am to 5pm [5]. Approaches to sampling wildlife products sold in black market shops are given by [5] as well as [6]. Computing this estimate however, depends on having a cluster sample data set in which all rhino horn purchases within each sampled cluster can be observed. It may be difficult to acquire such a sample.

**References**

1. Underwood FM, Burn RW, Milliken T. Dissecting the illegal ivory trade: An analysis of ivory seizures data. PLoS One. 2013; 8(10). Available: <http://www.ncbi.nlm.nih.gov/pmc/articles/PMC3799824/>. Accessed 21 June 2015.

2. Huh WT, Levi R, Rusmevichientong P, Orlin JB. Adaptive data-driven inven­tory control with censored demand based on Kaplan-Meier estimator. Operations Research. 2011; 59: 929-941.

3. Kariega. The Hanoi connection: Investigating rhino horn in Asia. 2014. Available: [www.kariega.co.za/blog/the-hanoi-connection-investigating-rhino-horn-in-asia](http://www.kariega.co.za/blog/the-hanoi-connection-investigating-rhino-horn-in-asia). Accessed 30 June 2014.

4. Cochran WG. Sampling Techniques, 3rd ed. New York: John Wiley and Sons; 1977.

5. Lam YKJ. Estimating the extent of illegal traditional Chinese medicine trade in Guangzhou, China using occupancy modeling*.* Masters Thesis, Imperial College Lon­don. 2012.

6. Regueira RFS, Bernard E. Wildlife sinks: Quantifying the impact of illegal bird
trade in street markets in Brazil. Biological Conservation. 2012; 149: 16-22.
