# Supplementary material for: Combating Rhino Horn Trafficking: The Need to Disrupt Criminal Networks
Source: PLoS One. 2016 Nov 21;11(11):e0167040. doi: 10.1371/journal.pone.0167040 (PMC5117767; doi:10.1371/journal.pone.0167040)
Supplement: S3 Text — (DOCX) [file pone.0167040.s003.docx]

**S3 Text**

**Cross-validation**

As reviewed in [1], data sets other than those used to parameterize an ecological model are needed to assess a model’s validity. When this is not feasible as in our case, [1] note that there are cross-validation techniques that can be used to mitigate the lack of generalizability of having only one data set with which to both parameterize a model and to assess its validity. Because our individual-based sub-model is a time series model of rhino abundance, we may make use of cross-validation techniques for validating time series models. The *rolling-origin-update* technique [2] computes a model-observation agreement statistic between model-predicted abundance at time $t+1$ using observations only up through time $t$ for $t$ set sequentially to a minimum, $m$ up through the last observation, $n$. Note that our individual-based sub-model, as with other population dynamics individual-based models, does not use any abundance observations at time $t$ in order to compute predicted abundance at the next time point, $t+1$. Therefore, in our use of the rolling-origin-update cross-validation technique, there is actually no “update” step. One cross-validation statistic is the mean absolute percentage error (MAPE), see [3]. Let $A_{i}$ be the actual, observed abundance at time $i$, and $F_{i}$ be the forecast abundance at time $i$. Then

$$\text{MAPE}=\frac{1}{n-m+1}\sum_{i=m}^{n} \frac{|A_{i}-F_{i}|}{A_{i}}.$$

Following this rationale, we compute the MAPE from Table 6. With the MAPE computation, we have (minimally) completed the first three of the five steps in the stepwise integrative validation of individual-based models proposed by [1].

**References**

1. Kubicek A, Joop F, Breckling B, Lange C, Reuter H. Context-oriented model validation of individual-based models in ecology: A hierarchically structured approach to validate qualitative, compositional and quantitative characteristics. Ecological Complexity. 2015; 22: 178-191.

2. Bergmeir C, Benítez J. On the use of cross-validation for time series predictor evaluation. Information Sciences. 2012; 191: 192-213.

3. Hyndman RJ, Koehler AB. Another look at measures of forecast accuracy. International Journal of Forecasting. 2006; 22: 679-688.
