# Supplementary material for: Combating Rhino Horn Trafficking: The Need to Disrupt Criminal Networks
Source: PLoS One. 2016 Nov 21;11(11):e0167040. doi: 10.1371/journal.pone.0167040 (PMC5117767; doi:10.1371/journal.pone.0167040)
Supplement: S4 Text — (DOCX) [file pone.0167040.s004.docx]

**S4 Text**

**Sensitivity analysis algorithm**

We evaluated how sensitive scenario outcomes are to misspecification of model parameter values. For this purpose we examined the results of the following sustainability-focused sensitivity analysis.

1. We define a persistent rhino population to be one for which the largest probability of extinction in the year 2045 for the Kruger National Park rhino population and the rhino meta-population on private ranches is less than 20%. Call this largest probability for the year 2045 the *extinction probability*.

2. For each purportedly non-sustainable strategic response scenario and each parameter, we search for a value that (a) is within an economically/ecologically valid interval, (b) is minimally different than the reference value, and (c) produces an extinction probability that is less than 20%.

3. If such a parameter value can be found for at least one of the purportedly unsustainable strategic response scenarios, then our main conclusion is excessively sensitive to possible parameter misspecification.

In agreement with the recommendations of [1] for conducting a sensitivity analysis of a stochastic individual-based model, we conducted our sensitivity analysis with an output measure (extinction probability) that is sensitive to the entire shape of the individual-based sub-model's rhino abundance probability distribution rather than assessing only the mean of that variable's distribution.

**Sensitivity analysis of rhino individual sub-model**

Our main conclusion of sustainability resulting when authorities compliment policies with disruption of trafficking syndicates while providing economic opportunities for people living next to parks is not unduly affected by possible parameter misspecification (Table 1): under reasonable ranges of parameter values (Table 5, main text), rhino extinction risk rises dramatically by 2045 if a policy is followed that excludes disruption of trafficking syndicates along with increased development of economic opportunities for people living next to parks. See above for the algorithm used to conduct this sensitivity analysis.

**Table 1**. **Sustainability-focused sensitivity analysis results.**

| Parameter | Status Quo | Pro-active  Protection | Demand Reduction | Horn Trade | Integrated Responses |
| --- | --- | --- | --- | --- | --- |
| Trader Learning Rate | 1.00 (all) | 0.67 (0.50) | 0.67 (0.50) | 1.00 (all) | 0.30 (0.3) |
| Consumer Reserve Price | 1.00 (all) | 0.56 (69877) | 0.33 (50124) | 1.00 (all) | 0.33 (69877) |
| Legal, Illegal Trader Maximum Capacity | 1.00 (all) | 0.22 (30) | 0.67 (40) | 1.00 (all) | 0.67 (40) |
| Life Expectancy | 1.00 (all) | 0.89 (35.5) | 0.67 (33.0) | 0.92  (35.5) | 0.44 (33) |
| Maturation Age | 1.00 (all) | 0.22 (3.50) | 0.33 (3.60) | 1.00 (all) | 0.22 (3.75) |
| Intercalving Interval | 1.00 (all) | 1.00 (all) | 1.00 (all) | 1.00 (all) | 1.00 (all) |
| Average Weekly Food Intake | 1.00 (all) | 0.67 (150) | 0.67 (150) | 1.00 (all) | 0.33 (160) |
| Maximum Energetic Budget | 1.00 (all) | 0.78 (5.25) | 0.67 (4.75) | 1.00 (all) | 0.33 (5.25) |
| Mean Energetic Budget | 1.00 (all) | 0.44 (3.75) | 0.20 (3.51) | 0.25 (3.51) | 0.22 (3.51) |
| Juvenile Energetic Budget | 1.00 (all) | 0.22 (3.50) | 0.20 (3.50) | 1.00 (all) | 0.22 (3.49) |

For each combination of parameter and non-disruptive strategic response scenario, the minimum extinction probability across the parameter's economically/ecologically valid interval of values is given. The accompanying parenthetical value is the parameter value at which this minimum probability occurred or the word ``all'' to indicate that the probability was constant across the interval.

Specifically, setting the anti-poaching effectiveness parameter, $p_{a}$ to values of 0.10 and 0.20 result in extinction probabilities that are greater than 20% when all other parameters are set to economically/ecologically valid values (Table 1); while the value 0.60 produces sustainable populations (Fig 3, main text). That these three values of$p_{a}$ represent real-world levels of anti-poaching effectiveness and lead to expected extinction probabilities, gives some credibility to the way in which we have modeled the effect of anti-poaching efforts on rhino population sustainability.

**Reference**

85. Bar Massada A, Carmel Y. Incorporating output variance in local sensitivity analysis for stochastic models. Ecological Modelling. 2008; 213: 463-467.
